# OSH related risks and opportunities for industrial human-robot interaction: results from literature and practice

**DOI:** 10.3389/frobt.2023.1277360

**Published:** 2023-10-30

**Authors:** Eva Heinold, Miriam Funk, Susanne Niehaus, Patricia H. Rosen, Sascha Wischniewski

**Affiliations:** Unit Human Factors and Ergonomics, Federal Institute for Occupational Safety and Health, Dortmund, Germany

**Keywords:** human-robot interaction, OSH risks and benefits, cognitive ergonomics, robotic systems, workplace automation, user expectations

## Abstract

Robotic systems are an integral component of today’s work place automation, especially in industrial settings. Due to technological advancements, we see new forms of human-robot interaction emerge which are related to different OSH risks and benefits. We present a multifaceted analysis of risks and opportunities regarding robotic systems in the context of task automation in the industrial sector. This includes the scientific perspective through literature review as well as the workers’ expectations in form of use case evaluations. Based on the results, with regards to human-centred workplace design and occupational safety and health (OSH), implications for the practical application are derived and presented. For the literature review a selected subset of papers from a systematic review was extracted. Five systematic reviews and meta-analysis (492 primary studies) focused on the topic of task automation via robotic systems and OSH. These were extracted and categorised into physical, psychosocial and organisational factors based on an OSH-factors framework for advanced robotics developed for the European Agency for Safety and Health at Work (EU-OSHA). To assess the workers’ perspective, 27 workers from three European manufacturing companies were asked about their expectations regarding benefits and challenges of robotic systems at their workplace. The answers were translated and categorised in accordance with the framework as well. The statements, both from literature and the survey were then analysed according to the qualitative content analysis, to gain additional insight into the underlying structure and trends in them. As a result, new categories were formed deductively. The analysis showed that the framework is capable to help categorise both findings from literature and worker survey into basic categories with good interrater reliability. Regarding the proposed subcategories however, it failed to reflect the complexity of the workers’ expectations. The results of the worker evaluation as well as literature findings both predominantly highlight the psychosocial impact these systems may have on workers. Organisational risks or changes are underrepresented in both groups. Workers’ initial expectations lean towards a positive impact.

## 1 Introduction

Interactive robotic systems have become a frequent occurrence in Europe’s workplaces over the last years. More and more workers find themselves working alongside a wide range of robotic technologies that assist them with their everyday tasks. These tasks can range from a robotic arm holding a heavy work piece for an industrial worker, to an automated guided vehicle which navigates the hospital hallways to deliver medicine (Kyrarini et al., 2021), tasks in the agricultural sector, like weeding, land preparation (Benos et al., 2023) or working more closely alongside humans assisting with the detection of fruit and vegetables, grasping and detaching (Vasconez et al., 2019). There are also robots working alongside waiters in restaurants (Lu, Zhang and Zhang, 2021). The areas of application are ever expanding. This way, robotic systems have contributed to creating more ergonomic and efficient work places (Jungmittag and Pesole, 2019). While the percentage of companies that use robotic systems capable of safe interaction with human operators is still comparatively low (Hämäläinen, Lanz and Koskinen, 2018), the International Federation of Robotics (IFR) reports an increase in annually installed robotic systems for the sixth year in a row. A trend which they predict to continue (Müller, 2022). The third European Survey of Enterprises on New and Emerging Risks (ESENER III) conducted by the European Agency for Safety and Health reveals that 28% of all human-robot interaction (HRI) applications were found in the manufacturing sector (Wischniewski et al., 2021). While other sectors are still gaging possible applications for these systems, the industrial sector already uses them actively and expands continuously in their use.

The relationship between occupational health and safety of workers and robotic systems can be multifaceted and complex. Industrial robots have traditionally been utilized for physically demanding tasks that can have negative effects on the health of a worker and may have a heightened risk of workplace accidents. Automating these tasks or larger parts of a manufacturing job through a robotic system has benefited workers by helping to prevent injuries (Haddadin et al., 2009) and adverse health effects that arise from working in hazardous conditions, such as musculoskeletal disorders caused by repetitive motions (Colim et al., 2020). However, if the robotic system is not used correctly and necessary standards for a safe interaction are not upheld, the technology may increase the risk for accidents (Yang et al., 2022) or introduce new hazards (Matthias et al., 2011). Even though, modern, interactive robotic systems are more commonly associated with their potential to remove workers from hazardous situations, and thus benefiting their safety and health (Kim et al., 2017), there is growing concern regarding the potential negative impact of human-machine interaction on the mental health of workers. Studies suggest that this relationship could have negative effects on workers’ wellbeing, while also becoming an additional source of stress in modern manufacturing workplaces (Robelski and Wischniewski, 2018; Körner et al., 2019). The increasing prevalence of robotic systems as a means of task automation can also increase stress (Venkataramani et al., 2020) and cause anxiety over potential job loss (Bhattacharyya, 2023). Moreover, it was found that implementing a robotic system to a workplace may trigger higher stress levels during the initial introduction (Wisse and Sleebos, 2016), and spike fear of job loss in the early days (Tuomi et al., 2021). Both effects seem to subside over time, bringing up the question how workers expect robotic systems to impact their work, not only in the short- but also in the long-term.

Evidently, the relationship between robotic automation and occupational safety and health (OSH) is complex, especially once the psychosocial implications are considered. While workers may see some OSH benefits related to automating a task, there may also be concerns regarding OSH related issues like job loss, the robots’ safety and their effect on workload (Wisse and Sleebos, 2016). The attitude and expectations of workers towards a technology can be a major contributor in the success of its implementation. Knowing about these factors before installing the technology offers the opportunity to adapt measures to address the mentioned issues. For this reason, existing studies, theoretical concepts and taxonomies are used in practical application to assess potential OSH related opportunities and risks when introducing robotic systems. One recently published report by EU-OSHA focusses on OSH impacts of advanced robotics in relation to the (semi-)automation of tasks. The authors of this report developed an OSH-factors framework for advanced robotics by defining dimensions that impact OSH during the introduction and use of robotic systems, as a means to assess possible risks and opportunities (Rosen et al., 2022).

This article focusses on whether and how these dimensions apply for the automation of physical task within the manufacturing industry. This will be assessed by considering both, a subsample of a systematic literature review as well as results from an evaluation of workers’ expectations within this field. We will analyse to what extent the OSH related dimensions and effects of robotic systems according to the OSH-factors framework for advanced robotics apply for the automation of physical tasks in the manufacturing sector. Moreover, we give an overview of the workers’ long- and short-term expectations towards the impact of robotic systems on their work and analyse whether workers primary expectations towards the system were positive or negative.

## 2 Industrial human-robot interaction and OSH

Robots capable working alongside humans are a comparatively new development, and represent only ion form of human-robot interaction. Onnasch Roesler, (2021) created a taxonomy to classify human-robot interaction in three distinct categories: coexistence, cooperation, collaboration. Coexistence describes an episodic encounter between humans and robots where the interaction is limited in terms of time and space, like passing a transport robot in the hallway. During a cooperation, robotic system and human worker work towards an overarching common goal. A robotic system performing a sorting task, while the worker uses the sorted parts to finish a work piece would be an example for this form of interaction. Collaboration describes an interaction in which both human and robot share an overarching task as well as sub-goals here. Their actions need to be coordinated and assigned consecutively. Human-robot collaboration is the most complex form of interaction. Industrial workers are at the forefront of jobs likely to come in contact with or get automated through robotic systems (Kadir et al., 2019; Dobra and Dhir, 2020; Gualtieri et al., 2021). Numerous sources report on robotic automation being used to automate tasks in the industrial sector (Gholamian and Ghomi, 2007; Iqbal et al., 2016; Enríquez et al., 2020). This includes tasks like pick-and-place or sorting tasks, holding work pieces, welding, assembly, paint spraying, packaging and arranging, cutting, moving, and sanding (Iqbal et al., 2016) as well as heavy lifting, precise physical activities and, specifically in a manufacturing context, the production of small volume assembly items in a high mix of products/precision works (Krzywdzinski, 2021). Traditionally, industrial robots operate spatially separated from shop floor workers. However, modern robotic systems are capable of working safely and efficiently alongside humans. This has allowed for new forms of human-robot interaction to arise. Robots which share an unfenced workspace with humans do require specified safety standards. Recommendations for collaborative robots (cobots) are summarized in the technical specification ISO/TS 15066 (Robots and robotic devices—Collaborative robots) (ISO, 2016).

There is evidence that these technologies impact the occupational safety and health of industrial workers. Both safety and efficiency are expected to increase through human-robot interaction (Gualtieri et al., 2021). Workers benefit from a decrease in physical strain through the automation of physically demanding tasks and increased safety of the work environment (Gualtieri et al., 2021). Recent publications on risk factors for human-robot collaboration also shine a light on emerging socio-technological risks, as well as on new ground with robot-centric ethical considerations and cybersecurity (Brex et al., 2022).

A growing number of workers now find themselves in the position that a robotic system has recently been introduced to their work place, or will be in the near future. This naturally triggers expectations towards the robot and the changes it brings to their work life. Not only regarding its impact on safety and health but more broadly speaking, its impact on their work overall, both long-term and short-term.

### 2.1 Worker expectations towards robotic systems

For an effective use it is advisable that a robotic system and the workers’ expectations towards it align. This typically relates to the robot’s features, functionalities or patterns of movement when it comes to direct interaction (Eyssel et al., 2011). However, looking at the larger picture, it is very rarely researched what general expectations there are towards how a robotic system will impact their workplace. In order to enhance the workplace interaction and long-term usage of the technology, it is important to consider the workers’ perspectives. This encompasses the expectations of workers prior to the robot’s introduction, which should not be limited to only its functionalities, but the larger impact that is expected. Without considering human factors during the implementation, however, the introduction of such systems tend to fail (Fletcher and Webb, 2017). Few publications address general expectations towards robotic systems from a workers’ perspective, and equally few investigating the workers’ specific expectations towards OSH with regards to the robotic system (Wurhofer et al., 2015; Aaltonen et al., 2017; Elprama et al., 2017; Kildal et al., 2018; Willems et al., 2023). This article is therefore an enrichment to the current scientific discourse, as industrial workers’ expectations were assessed and analysed using global categories as proposed by the OSH-factors framework for advanced robotics (Rosen et al., 2022).

One study which does address workers’ expectations in the manufacturing sector is conducted by Wurhofer et al. (2015). They studied workers’ expectations prior to the introduction of robotic systems to a semi-conductor factory and accompanied the workers throughout the process. Within their study, statements of uncertainty as well as scepticism and rejection were the most frequent. However, positive expectations were also present. While OSH relevant factors were mentioned, it was not in the foreground of workers’ expectations. These results align with further studies on the topic. Workers expect interactive robotic systems to lighten their mental and physical workload (Elprama et al., 2017). Other studies found, that workers expect physical workload to decrease, and safety to increase, however, they also expect their workload to increase along the robot’s productivity (Aaltonen et al., 2017; Kildal et al., 2018). Kildal et al. (2018) asked potential robotic users from robot related industries for their expectations towards the technology. The participants expected the impact of robots as a whole to be positive (productivity, quality, competitiveness, safety, costs and working conditions). The most negative expectations were centred on job loss (Kildal et al., 2018).

Within this limited body of studies, we see that workers tend to have mixed expectations towards the change robotic systems might bring. While the physical changes are primarily expected to be positive, both psychosocial and organisational changes that are brought up lean towards the negative. Additionally, a varied time perspective is rarely explored. Instead, in many cases, a timeframe is not defined and the studies are focusing on the most immediate expected changes. Incorporating short- and long-term expectations from workers and interpret them within the dimensions of OSH impacts in advanced robotics yields an opportunity to broaden the understanding about the most prevalent factors from a worker’s perspective, in order to facilitate successful long-term use of the technology.

## 3 OSH-factors framework for advanced robotics

**FIGURE 1:**
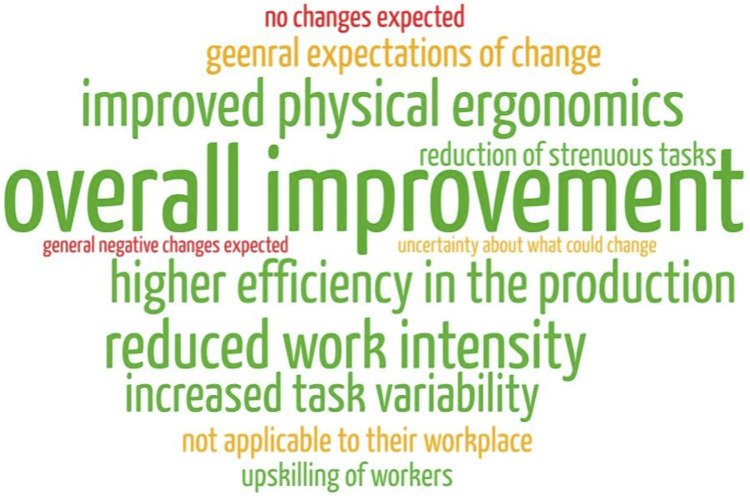
EU-OSHA Framework of OSH relevant dimensions for the introduction and use of robotic systems.

To provide meaningful advice for the implementation of robotic systems in the workplace, all relevant components of a work system should be considered. This includes the physical and psychosocial context as well as the social and organisational work environment (Leka and Jain, 2010). In a recently published report on the OSH impacts of advanced robotics for the (semi-)automation of tasks, the authors present an overview of OSH relevant dimensions (Rosen et al., 2022). They utilize three foundational categories (*physical*, *psychosocial* and *organisational*) and subdivide them into eight sub-categories (physical alteration of the workplace, function allocation, task design, interaction design, operation and supervision, introduction process, change management, training) representing areas of OSH relevance in this context (Figure 1). Based on an extensive literature review, the authors found that these facets may result in different positive or negative OSH outcomes. The presented categories may all effect OSH during the introduction and interaction with the robotic system. The framework provides a comprehensive categorisation of relevant aspects and has proven to be an adequate framework to assess OSH related risks and benefits while taking specific task and technology characteristics into account. It was created with considerations regarding automation type as well as relevant OSH characteristics, in order to suite the automation of tasks through robotic systems. This sets it apart from non-robot specific OSH frameworks for the automation of tasks for a wider variety of technologies and the automation of tasks more generally (e.g., Nickel et al., 2020). While this framework presents one possible set of categories to base an analysis on, there are others attempting the same thing. Berx et al. (2022) also created a categorisation based on a systematic literature review, which resulted in five overarching groups (Human, Technology, Collaborative Workspace, Enterprise and External). Content wise these categories are parallel the OSH-factors framework by Rosen et al. The category External may be especially useful, for research that aims to include the wider context of robot use. The following section provides an overview of the different dimensions and their subcategories as described in Rosen et al. framework. These form the foundation of a content analysis on whether and how the OSH related dimensions and effects of robotic systems apply for the subset of physical task automation in the manufacturing sector as well as their suitability to categorise worker expectations.

### 3.1 OSH dimensions

#### 3.1.1 Physical

The automation of tasks via robotic systems is especially associated with changes in the physicality of tasks or the working environment. Changing a physically straining task to be supported by a robotic system can impact physical OSH. The actual OSH benefits and risks that an advanced robot brings to a workplace is highly dependent on the use case and technology and are not limited to physical effects. For example, removing a worker from a dangerous environment does decrease the risk of physical harm, however it may also lighten the psychological stress associated with working in a dangerous surrounding.

##### 3.1.1.1 Physical alteration of the workplace

Robotic systems are predominantly used to automate physical tasks, and thereby change the physical workspace and job demands of workers (Rosen et al., 2022). Robots may also physically support workers in tasks that cause repeated physical strain (Kyrarini et al., 2021), possibly reducing work-related musculoskeletal pain and injuries. However, the introduction of advanced robotics may also introduce new OSH risks to a workplace, like collisions. In order to not introduce new physical risks, contact avoidance measures, motion planning, and sensor systems play a significant role in ensuring the operators’ safety.

#### 3.1.2 Psychosocial

Psychosocial effects include a range of phenomena relating to a worker’s mental, emotional, or social state. Based on the dimensions of the OSH-factors framework for advanced robotics (Figure 1), these four categories are most likely to expect changes due to the implementation of robotic systems at workplaces. Depending on how these categories are executed, they may affect workers strongly on a psychosocial level.

##### 3.1.2.1 Function allocation

Function allocation in task automation involves determining the division of tasks between humans and robotic systems based on the specific task requirements (Robelski and Wischniewski, 2018; Tausch et al., 2020). While static task allocation is a common approach, as robotic systems become more flexible and capable, task scheduling becomes more dynamic. The resulting distribution of tasks holds implications for occupational safety and health regarding various psychological factors, including but not limited to perceived process control, mental effort, fairness, task identity, acceptance, flow, and self-efficacy (Tausch et al., 2020).

##### 3.1.2.2 Task design

The process of function allocation results in direct consequences for the task design. How a task is designed may change once a robotic system is installed in the workplace. If tasks are predominantly designed around robotic performance and speed, it can result in the workers’ pace being determined by the robotic system. This may result in negative psychosocial effects, including but not limited to emotional exhaustion, nervousness, irritability, worse mental wellbeing, and reduced job satisfaction (Robelski and Wischniewski, 2018; Rosen and Wischniewski, 2019). A concern regarding the introduction of advanced robots into workplaces and the changes they trigger in task design is possible work intensification, as described in the Job-Demand-Resources Model (Demerouti et al., 2001). It might manifest as increased work demands and higher expectations placed on workers, a quickened work pace or an increased quantity of work. It may also manifest as reduced autonomy or the expectation to multitask.

##### 3.1.2.3 Interaction design

The interaction between workers and robotic systems can influence a number of OSH related factors. This can relate to, among others, the way they handle interaction, as well as how transparent and comprehensive the interaction is perceived by the user. Another aspect of interaction design that needs to be considered in HRI, is the transparency of the system. When transparency is lacking and the operator is left without the necessary information to follow the underlying reasoning, a robot might be perceived as unreliable (Kim and Hinds, 2006). However, more information is not always better. An overabundance of information might even decrease transparency, leading to difficulties in selecting crucial information by the worker (Finomore et al., 2012). Furthermore, the interaction with the robotic system should be designed in such a way, that its users perceive the system as safe. Transparency is one of several factors influencing this, alongside familiarity, predictability, sense of control and trust (Akalin, Kristoffersson and Loutfi, 2022).

##### 3.1.2.4 Operation and supervision

Operation and supervision refers to the management and oversight of the day-to-day activities and processes when working with a robotic systems. A number of topics fall into this category such as the allocation of resources and monitoring of performance. One psychosocial factor that should be taken into account during the introduction of robotic systems to the workplace is the attitude and experience towards and with robots present in the workers. A lack of familiarity may shape initial attitudes (Sanders et al., 2019). Moreover, it was found that trust and acceptance tend to increase as workers are exposed to the systems (Hancock et al., 2011) while negative attitudes decrease over time (Nomura et al., 2011). The fear of job loss is one of the most thoroughly researched topics in the context of robotic automation (McClure, 2018) and given that approximately 40% of workers will experience significant changes in their work due to the introduction of robotic systems to the workplace (Pouliakas, 2018), it represents another important psychosocial factor. More so in light of the evidence that job insecurity is linked to the risk or presence of depression, anxiety and emotional exhaustion, as well as to low satisfaction with life (Llosa et al., 2018).

### 3.2 Organisational

The effects of introducing a new technology to a workplace can reach further than the physical or psychosocial aspects of OSH. In some cases, it leads to OSH related organisational changes, or the introduction itself needs to be preceded by specific processes to maximize the OSH benefits of the technology.

#### 3.2.1 Change management

Change management in a company refers to the structured approach aimed at preparing and implementing organisational changes. Effective communication and active participation are crucial for a successful introduction of a new technology. Informing and involving employees in workplace changes can have positive effects on acceptance and enhanced commitment (Bordia et al., 2004). Change management encompasses the company culture around the process and how they deal with problems that may arise. If change management fails for a technology that was intended to bring OSH benefits to workers, they may now not experience these positive effects. Unsuccessful change management may also result in feelings of uncertainty, stress (DeGhetto et al., 2017), while successful change management may increase them (Chien, 2015).

#### 3.2.2 Introduction process

The introduction process of a new technology falls under the umbrella of change management. It is, however, more specific to the technology being implemented. It includes the involvement of all stakeholders in the process, but also pilot testing, risk assessment, training, as well as pre and post assessments. Factors like proper risk assessments are vital to OSH. However, the involvement of effected parties can be influential on OSH as well. Communicating future changes to employees can reduce feelings of uncertainty towards the rationale behind the change and promote change supportive behaviour (Bordia et al., 2004). Employee participation and involvement play a part in the acceptance and the implementation and outcomes of technological transformations in the workplace (Krutova et al., 2022). Increased worker participation also correlates with better risk assessments and more effective preventive measures, especially concerning psychological strain (Popma, 2009).

#### 3.2.3 Training

For many workers, advanced robotic systems are still a new technology with which they have little to no prior experience. Changes in the work equipment or work routine might incite the need for workers to acquire new skills or change their overall skill portfolio, some skills even might become dispensable. Some organisations predict that the automation of tasks will lead to skill polarization in the workplace, where available jobs are extreme in complexity, either very high or very low, with little available middle ground (ILO, 2017). Specialized training specific to the robotic technology and work situation may be necessary to ensure effective and safe use of the systems. While this offers the potential for workers to perform more interesting tasks, continuous learning may also pose a new cognitive strain on the workers.

## 4 Methodology

The data for this publication was collected via two methods. The data sources were a subset of studies from a systematic literature review and a worker survey, the results of which were then subjected to qualitative content analysis and interrater reliability using Fleiss kappa was calculated. All calculations were performed with IBM SPSS Statistics Version 29 (IBM, 2022).

The original literature review was performed as part of a larger research project funded through the European Agency for Safety and Health at Work. The original review aimed to create an overview of policies, research and practices in relation to advanced robotics and AI-based systems for automation of tasks and occupational safety and health; which is a much wider scope than this research paper addresses. Part of the author team of this article was involved in the creation of the framework used in this article. The worker survey was part of the EU-funded project “Socio-Physical Interaction Skills for Cooperative Human-Robot Systems in Agile Production” (SOPHIA, Funding Agreement No. 871237).

### 4.1 Systematic literature review

**FIGURE 2:**
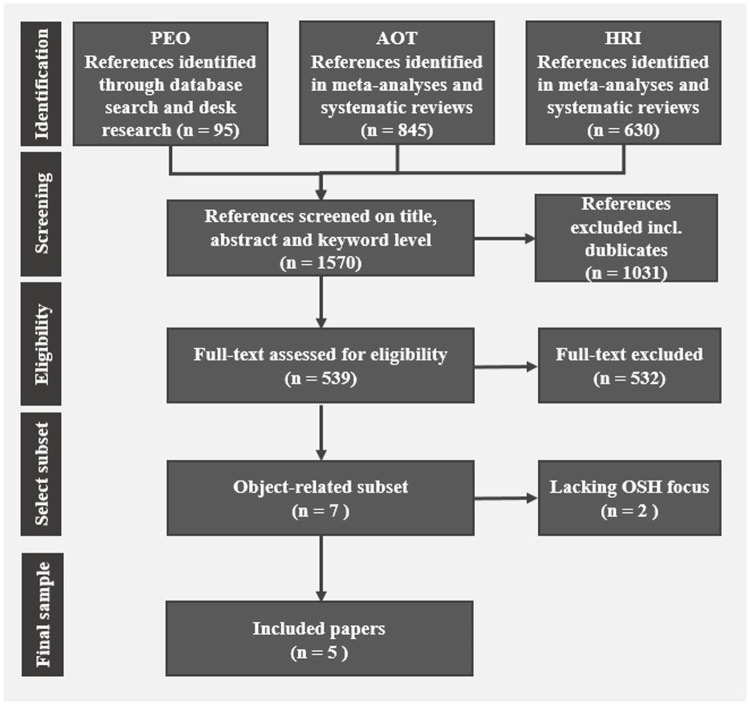
Literature review process.

Two systematic literature reviews were conducted with a focus on systematic reviews and meta-analysis only, examining human-robot interaction and the automation of tasks within EU-OSHA’s original publication (Rosen et al., 2022). Their publication focuses on the central question where current research activities regarding advanced robotic and AI-based systems lie, whereas our analysis focusses on the question what OSH implications are addressed in the manufacturing sector with regards to advanced robotics specifically. We aim to investigate whether and how OSH related dimensions and effects of robotic systems apply for the subset of physical task automation in the manufacturing sector as well as their suitability to categorise worker expectations. Hence, we specifically selected the subset of publications that focused on the automation of physical tasks in the manufacturing sector, which featured OSH relevant findings. The selection process from each search is illustrated in Figure 2. The literature review was conducted in scientific and complementary databases (IEEEexplore, Ebscohost, WebOfScience, PubMed, and Google Scholar). An additional systematic literature review focused on the automation of tasks, independent from any specific technology. Supplementary literature was obtained through additional desk research using the same data bases, in order to elaborate on the previous results. A comprehensive combination of search terms was developed following the PEO-scheme (Population—Exposure—Outcome). The complete search strings, as well as a more detailed description of the review process can be found in the publication Rosen et al. (2022). The review only included publications meeting set criteria. They had to be meta-analysis or literature reviews, focussing on human-robot interaction. For the initial quantitative reporting, they did not have to include OSH specific results, for the second step of analysis in Rosen et al., only publications with OSH related insights were included. Rosen et al., aimed to create an overview of the state of research, hence they did not limit the sector or type of task the publications had to address, as long as they included a work-related application of an AI-based system or advanced robotics. For our publication the selection was narrowed down, significantly, by enforcing additional selection criteria. For our analysis, five publications met the selection criteria of focusing on the automation of physical tasks in the manufacturing sector, or being applicable to this field, while containing OSH relevant outcomes (Prewett et al., 2010; Kadir et al., 2019; Dobra and Dhir, 2020; Rauch and Dallasega, 2020; Ötting et al., 2022). Major outcomes from these studies were extracted to be analysed in this study. An overview of the included studies can be found in Table 1.

**TABLE 1:** Overview of the included studies.

Author	Year	Study	Number of primary studies	Technology	NACE-sector
Dobra and Dhir	2020	Technology jump in the industry: human-robot cooperation in production	87	Industrial robots	Manufacturing
Kadir et al.	2019	Current research and future perspectives on human factors and ergonomics in Industry 4.0	90	Industrial and collaborative robots	Manufacturing
Prewett et al.	2010	Managing workload in human-robot interaction: A review of empirical studies.	113	Robotic systems	Manufacturing
Rauch et al.	2020	Anthropocentric perspective of production before ad within Industry 4.0	58	Industrial robots	Manufacturing
Ötting et al.	2022	Let´s work together: A meta-analysis on robot design features that enable successful Human-Robot interaction at work	81	Industrial robots	other

### 4.2 Worker survey

To assess the workers’ perspective, we performed a survey as part of the SOPHIA project that included workers from three European companies. They were asked about their expectations regarding changes, benefits and challenges of robotic systems at their workplace. All three companies are part of the manufacturing industry but differ in size, core business and country of origin (Germany, Netherlands, and Slovenia). Twenty seven workers were asked about their expectations regarding a robotic system that was planned to be implemented at a workstation at their company. The number of potential participants was limited in order to survey workers with a high level of experience and therefore expertise at the selected workplace. All the workers who took part in the survey had at least 1 year of experience of working at the chosen workstation. The workstations considered were selected by the companies after identifying a suitable task that could be facilitated by a robotic system. All selected workstations involved repetitive tasks that had recently been performed manually: the unloading of steel laminates after an annealing line, the manufacturing process of gear cutting and the attachment of a rubber seal to the car body.

The questionnaires and surveys were conducted over a period of 2 weeks in small online groups, due to the pandemic restrictions in autumn 2020. The focus was set on workers’ expectations regarding aspects related to the usability of the respective robotic system. Ethical approval was obtained beforehand, and a data protection declaration was carried out and approved by the organization’s data protection officer. Participating in the study was voluntary during their working hours. Each round of the survey lasted approximately one to one and a half hours. The workers completed paper and pencil versions of the survey, which were returned by post to the responsible scientists for analysis.

The aim of the study was to gather data on workers’ expectations before developing a specific robotic system, they were asked to imagine working with a robot that could perform various tasks to support their daily work. To give the employees a better idea of the intended scenario, they were shown a picture of the intended workstation and a picture of the chosen base platform for the development of a robot as an anchor example for the collaborative robots. In one company the robotic system had been introduced a few weeks before the survey, so the workers were already familiar with it. Therefore, the participants were asked to imagine that the functionality of a robotic system would exceed the level of the current system, with the aim of gathering expectations of robotic systems from a more general point of view. Besides well-established questionnaires on system usability, acceptance, strain and job control, open format questions were included, asking the workers to express their initial expectations towards the changes brought by the robot: How do you think your task will change by using the robot?; Do you expect benefits from using the robot (in the short- and long-tern)?; Where do you see potential problems when using the robot (in the short- and long-tern)? For this article, we analysed theses open-ended questions. By this, we aimed to gauge if the primary association with the technology from workers is positive, negative or neutral. Their statements were not limited to a specific number of expectations to express or to OSH related changes.

### 4.3 Content analysis

Central questions to the analysis were whether and how the OSH dimensions and effects laid out in the OSH-factors framework for advanced robotics apply for the automation of physical tasks in the manufacturing sector. Furthermore, we wanted to find out if the results of the literature match the expectations of the surveyed workers and if tendencies (positive or negative) can be identified for the respective categories.

To elevate the collected statements from both literature and the worker survey, we performed a Qualitative Content Analysis (QCA) (Mayring and Fenzl, 2019), which is a commonly used methodology to analyse qualitative data. QCA concentrates on describing and reducing or summarizing the collected material focussing on the particular analysis object as well as the material context (Mayring, 2014). Since objective criteria, common in quantitative studies to assure a high research quality, are not easily transferable to qualitative research, it is important to focus on methodological consistency (Corbin and Strauss, 2014). Equally important is iterative data collection and analysis, enabling a comprehensive consideration of different perspectives and contexts. Ideally, there is a balance between following a systematic approach and discussion-based consensus (Strübing, et al., 2018). Therefore, decided on a structured analysis approach for which the underlying code were the categories of the framework (*physical*, *psychosocial* and *organisational*) including its subcategories with the above written descriptions (Figure 1). These deductive categories provided the guideline for the initial analysis.

On this basis, three independent raters categorised both the literature and worker statements to relate to either *physical*, *psychosocial* or *organisational* changes. The raters were part of the research team and are considered experts in the field of robotic systems and human factors with academic backgrounds in psychology, sociology, cognitive science and computer science. In a second step they also assigned each statement to one of the OSH-factors framework subcategories. Furthermore, they had the option to withdraw a statement from the selection should it not contain information that could be attributed to a category (for example, “*I do not think the robot could do my job successfully*”). Once each rater had independently categorised the statements, the results were compared and the researchers discussed any points of controversy. When all raters assigned a statement to the same primary category it was classified as an agreement. An overlap of two out of three raters in the subcategory was also seen as an agreement on the subcategory level. Any remaining disagreements were discussed and resolved among the raters. All statements that were not made in English were translated using DeepL, and translated back, to reduce loss of information. In total, 16 statements made by workers were excluded from further analysis (plus three who choose not to answer the question). During the process of discussion, the raters noticed repeating patterns in the assignment of categories. Hence, they decided to form new, inductive subcategories on the basis of the existing framework.

## 5 Results

In the following section, we present the results of the content analysis. The OSH-factors framework’s categories were considered as a basis to assign major insights of the selected research papers into the categories, where possible. The first section presents selected results from the literature review, while the second, greyed, section presents exemplary replies of the workers (Table 2). It was possible to categorise both, the worker statements as well as the excerpts from literature, using the primary categories of the OSH-factors framework for advanced robotics. Several statements, however, were categorised as too ambiguous to be assigned a definitive subcategory by the raters. The sample included only 25 male and 2 female participants, of whom 72% were working directly at the production line, 24% were craftsmen and one person was in middle management. In order to ensure the anonymity of the participants, data on age was not collected.

**TABLE 2:** Categorisation of statements.

Physical		
Subcategory	Risk	Opportunity
Physical alteration of the workplace	- Close human robot collaboration evokes safety concerns	
	- Residual risk/unreliability cannot be eliminated completely	- A [robot] cannot always avoid colliding with humans. Safety sensors reduce the force of impacts and stop the robot movement when bumping into a human, but the residual risk remains
	- Some operators experience mental stress because of safety concerns during close collaboration with robotic systems	- Robots can help compensate physical limitation of human workers
	- […]	
		- “Ergonomic improvement, increase of occupational safety”
		- “Less physical load as a result of which in an older age you have fewer complaints or would never get worse from them”
	- “Combining human and robot safety at work and detection of border pieces”	- “Less suffering joints and muscles”
	- “Space around the machine, weight of the products”	- “No more heavy physical work”
	- “More space by the machine” (room)	- “Preservation of your physical condition. Less physical complaints”
		- “Protection of body and psyche”
		- […]
Psychosocial		
Function allocation		- “Multiple machines save more time”
Task design		- “Facilitate/simplify the work”
		- “Less repetitive work and therefore less work pressure”
		- “Makes work more interesting”
		- “More time left for maintenance and other important things”
		- “Setting up the robot cost time in the beginning, but later you benefit from it because the programs already exist and you can therefore do other things”
		- […]
Ambiguous (Task design or Function allocation)		- Robots and collaborative robots can perform easy, repetitive, monotonous and straining manual tasks (dull tasks) instead of humans
		- Hybrid production systems [incl. robots] can bridge the gap between humans and machines abilities
		- Cobots can perform unsafe, repetitive, or boring tasks so workers can perform other more value-added tasks
Interaction design	- Working with an advanced socio-technological system can result in a degree of uncertainty	- Autonomous robots might be able to identify and adapt to a worker’s individual strengths and needs
	- Audio feedback while controlling a multi-robot set up increases reaction time	- The interface design of a robotic system can significantly influence performance, cooperation and satisfaction, by increasing feature visibility and giving feedback
	- Lack of confidence in sensory systems for physical contact [during HRI]	- Minimize injury through viscoelastic coverings, mechanical absorption systems, lightweight structures and collision detection systems
		- […]
	- “High error rate, complicates handling”	
	- “Perishability of the robot and its repair	
	- the consequences of a delay in production”	- “The simple handling”
	- “I foresee many technical problems in the human-machine-robot collaboration.”	- “That it works”
	- “Prone to failure, acceptance of the workforce”	
	- […]	
Operation and supervision	- Residual risk/unreliability cannot be eliminated completely	- Reliable automation can improve operator performance
		- Automating tasks through robotic automation might lessen operator workload, if the technology is reliable
	- “Older” persons have fear of failure, problems of understanding”	- “Increase work performance”
	- “Elimination of personnel by machinery use”	- “Increasing productivity through daily operation in the service, healthcare”
	- “Replacement of employees”	- “More productivity”
	- […]	- “More profit for the company”
Ambiguous (Interaction design/Operation and supervision)	- As system complexity increase, so might the cognitive workload of operators	
	- Controlling more than two robotic systems can decrease performance and increase error rate	- Effective HRI is achieved by considering both humans and robots [abilities]
	- “Difficulties in examining the use, not related to the technology”	- The mental status of the human partner plays an important part in the collaboration […]. [It is proposed to] adjust the human workload according to the stress level of the operator
	- “Service and manipulation in production”	
Organisational		
Training	- Cognitive overload of workers [due to constant need for learning]	
	- [Industry 4.0 incl. robots] is driven forward more quickly than training and education institutes are able to adapt the qualification profile of existing and future workers	
	- “Knowledge when using it”	
	- “Problem in robot learning (use)”	
Change management	- Without effective human leadership, and material resources operators will struggle to be effective	- Robots will support demographic and diverse team structures
	- Fear, that increasing digitization will result in a large wave of unemployment	- Participation, communication, manager support, training, worker empowerment and existing process [are process enabler when introducing a robotic system]
	- Union membership, awareness of process complexity, manual process variability and [scarcity of] resources [are barriers]	
	- “Destruction of many jobs, chance for a basic income”	- “When we manage to implement it in the environment it certainly picks up the acquisition of the yield, the work done”
		- “Not in the short-term. Think that a lot of time is needed for the work on the shop floor”
Introduction process

### 5.1 Interrater reliability

Fleiss’ kappa was calculated to determine if there was agreement between the raters on the primary categories assigned to the statements from literature and the worker survey. The base categories were taken from the OSH-factors framework, namely, *physical*, *psychosocial* and *organisational*. For the statements extracted from literature, the kappa regarding the primary categories was (κ) = 0.624, a 95% confidence interval (CI) between 0.478 and 0.770. The result was statistically significant (*p* < 0.001) and represents a substantial strength of agreement between the raters. For the worker statements Fleiss’ kappa (κ) = 0.755, a 95% confidence interval (CI) between 0.679 and 0.835. The result was statistically significant (*p* < 0.001) and represents a substantial strength of agreement between the raters (Landis and Koch, 1977).

### 5.2 Categorisation

After the initial round of analysis which resulted in the categorisation (Table 2), it became apparent, that the categories of the framework are quantitatively and qualitatively addressed to varying degree in the workers’ replies as well as in the literature statements, and present a varied image towards the risks and opportunities associated with robotic systems in industrial workspaces. Regarding the category of *physical* factors, and its only subcategory *physical alteration of the workplace*, physical closeness to the machine was a concern, however, literature indicates that only a residual risk of physical complications remains. This is strengthened by the listed opportunities, which highlight that new, advanced sensors allow safe and close interaction. Workers highlight the reduction of physical load and health complications, especially in the long-term (“Less physical load as a result of which in an older age you have fewer complaints”). Regarding the category of *psychosocial* factors, the raters assigned most statements unanimously to the primary category, however in the subcategory there were two clusters of statements that were labelled as too ambiguous to be assigned to one of the four subcategories. *Function allocation* was assigned near to no statements from either workers or literature. *Task design* only contained opportunities or positive expectations workers, no statements from literature. Workers expect their tasks to become “less repetitive” and “more interesting.” Several statements from literature were assigned to a distinct subcategory, as they could reasonably describe *function allocation* or *task design*. Content wise however, they mirrored workers expectations (e.g., Cobots can perform unsafe, repetitive, or boring tasks so workers can perform other more value-added tasks). These statements contained facets of both how the task would be effected as well as who would perform it. The raters discussed this overlap and came to the consensus, that while *task design* and *function allocation* are distinguishable in a theoretical context, when analysing workers’ experiences and expectations it is a too high level of detail to apply. However, the importance of both topics was recognized by the raters, so the researchers propose to combine the two categories into shared one called “*function allocation and task design.*” A similar situation emerged when it came to the categories of *interaction design* and *operation and supervision*. Depending on the perspective applied to the statements, both categories were applicable and assigned by at least one rater. A statement from the workers perspective can be interpreted to relate more to the expected interaction with a technology, whereas from a company perspective, it would be more related to *operation and supervision*. Hence, the categories were ultimately combined into one group called “*interaction design, operation and supervision”.* Statements that were categorised as related to primarily *interaction design* from literature, focussed primarily on how interface and interaction modalities effect the interaction, both in a positive and possibly negative direction. Workers mainly anticipated malfunction from the robot. In the category *operation and supervision*, literature highlighted reliability or unreliability as a determining factor for the effectiveness of a robotic system in the industrial sector. Workers positive expectations leaned towards increased productivity, while they negatively anticipated job loss, and demographic challenges with regards to learning new, robotic related skills. The *operation and supervision*, literature highlighted reliability or unreliability as a determining factor for the effectiveness of a robotic system in the industrial sector. Workers positive expectations leaned towards increased productivity, while they negatively anticipated job loss, and demographic challenges with regards to learning new, robotic related skills. The *organisational* factor and its subcategories were the least populated among the three. Both literature and workers only listed risks regarding *training*. They both described the challenges of re-education and the cognitive demand this poses on workers. *Change management* was represented nuanced in both sources. Literature stressed ineffective leadership as curtail in enabling people to work effectively with the technology, and point out the potential for a more inclusive workplace. Workers perspectives included statements addressing a potential development towards universal income and more long-term developments on the shop floor. Noticeably, neither literature nor worker statements addressed the *introduction processes*.

### 5.3 Changes, short- and long-term expectations by workers

The survey asked workers about their general expectations of changes. By keeping the initial question of this set open, we aimed to gauge if the primary association with the technology from workers is positive, negative or neutral. These results can deliver an indication if workers had a primarily positive, neutral or negative outlook towards the changes brought by the technology. However, focusing too strongly on the quantity of the expectations named might result in a skewed representation, as workers were not limited to a specific number of expectations to express. To provide a comprehensible overview, statements relating to the same general topic (e.g., “*lifting fewer heavy objects*” and “*work will become less physically demanding*”) were summarized under group names presented below (Table 3). After every group we provide an indication how many statements were included in it.

**TABLE 3:** Initial expectation of change from workers.

Positive	Neutral	Negative
- Reduced work intensity (2)		
- Improved physical ergonomics (5)		
- Increased task variability (2)	- General expectation of change (2)	
- Reduction of strenuous tasks	- Uncertainty about what could change	- No changes expected
- Overall improvement (7)	- They do not see a robotic system as applicable for their workplace (2)	- General negative changes expected
- Higher efficiency in the production (5)		
- Upskilling of workers		

**FIGURE 3:**
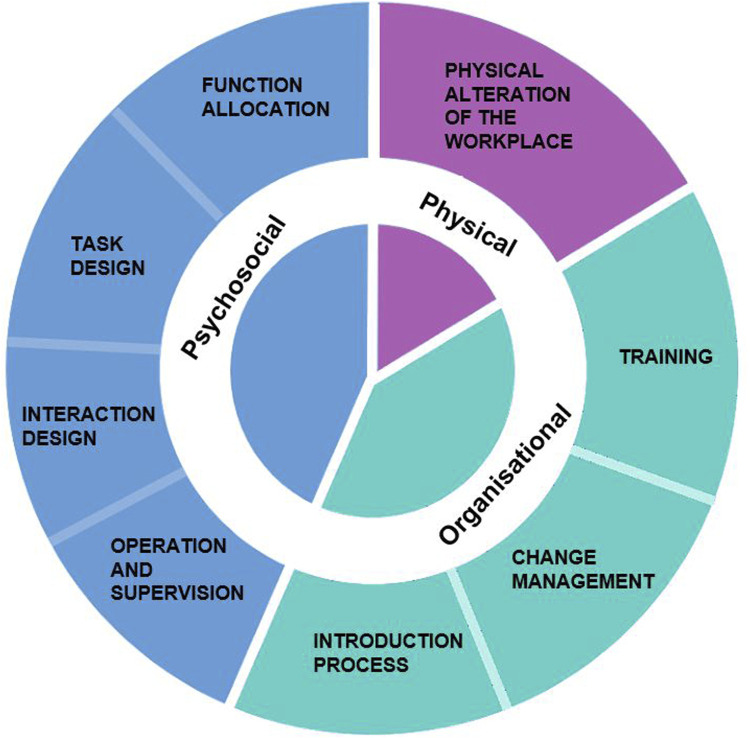
Visualisation of positive, neutral and negative expectations towards robotic systems.

To further illustrate these finding, Figure 3 displays the named positive, negative and neutral expectations towards robotic systems. The size of each word is relative to the frequency the category was mentioned. Green writing indicates positive aspects, yellow neutral and red negative aspects. Next, workers were specifically asked what types of effects they expect the robotic system to have in the short- and long-term. Differentiating between the immediate and continuous impact of a technology may grant insight into a more layered opinion of workers on the technology. Table 4 provides an overview of the workers responses to both the short- and long-term category. Participants were again not limited in how many expected benefits or problems they could name.

**TABLE 4:** Short- and long-term risks and opportunities expected from workers.

Short-term	Long-term
- Technological failures (2)	
- Unclear task allocation	
- Reduced physical workspace (3)	- Job loss (4)
- Reduced product quality	- Decreased productivity
- High error rate (3)	- Increased monotony
- Stress (2)	- Malfunctions and errors
- Low acceptance	
- (Lack of) training (2)	
- Safety concerns	
- Increased job control	- Increased task variability (2)
- Reduced work intensity	- Improved time control
- Increased productivity (2)	- Improved physical ergonomics (7)
- Reduced monotony	- Improved physical and cognitive ergonomics
- None (2)	- Reduced long-term health complications (3)
- Improved wellbeing	- Economic growth
- Reduced work intensity	- Job transformation (2)
- Improved physical ergonomics (3)	- Increased productivity
- Improved cognitive ergonomics	- Overall improvement (2)
- Overall improvement (4)	

## 6 Discussion

The introduction of advanced robotic systems at an industrial workplace can change working conditions drastically for the employees. These changes can permeate aspects regarding physical, psychosocial and organisational factors concerning, but not limited to, occupational safety and health. While it is vital to consult research on possible effects such a technology can have on workers, it is also important to assess the expectations of those who will be directly affected by the technology. Using existing frameworks like the OSH-factors framework for advanced robotics can provide a basis for comparison and further analysis.

### 6.1 Content analysis

The selected literature focusses on OSH related risks and opportunities for industrial human-robot interaction. All five studies contain various outcomes that describe how OSH is affected by advanced robotic systems in an industrial setting. The results show that these studies cover a vast variety of factors. When it comes to opportunities, the analysed literature does not provide any insight regarding the categories of *task design, organisation and supervision* as well as *training*. With regards to possible risks the category of *change management* was underrepresented. Furthermore, the category *introduction process* was neither addressed regarding any opportunities nor risks. The analysed literature only presents a small, yet specific subsample of all available literature on robotic systems. The present distribution may still be used as an indicator of areas which are in need of more focused research in the future. The worker statements were similarly distributed, with the greatest focus on physical effects followed by how their direct task might change. The fewest statements were assigned towards organisational aspects. Possibly, because the effect of organisational changes is the furthest removed from their area of influence. Even though neither literature nor worker statements addressed the introduction processes, the topic is of major relevance. The distribution supports the findings of Berx et al. (2022), where Technological and Human related OSH factors were noticeably more present in their reviewed literature as in the Enterprise category. The underrepresentation of the organisational category on the workers’ side might be due to the framing of the survey. The questions were phrased in such a way that it could be assumed, the robotic system had already been installed and the introduction process finished. Future studies could consider investigating workers expectations towards the introduction process specifically, to gain insight on the needs and expectations of workers during this time of change. When focussing on the content both sources provided, we can see that they align in some categories, while others focus on different aspects of the topic.

During the categorisation of statements, a few points of discussion came up. One that was repeatedly raised between the raters, was the perspective under which any given statement should be analysed under. Depending on that, the category that was considered fitting for a statement changed among the rater. For example, “*Personalized, adapting systems could result in continuous monitoring, which raises concerns for privacy*” was categorised as applicable for *interaction design*, *operation and supervision*, and *change management*. The categorisation depended on where the focus was being set and if they were seen to relate to the worker perspective, developers’ perspective or the company’s perspective. This change of category depending on the mikro- or meso-view of a working situation poses a challenge for this type of content analysis.

The OSH dimensions and effects laid out by the OSH-factors framework for advanced robotics largely apply for the automation of physical tasks in the manufacturing sector regarding advanced robotic systems, especially when looking at the three main categories proposed. However, as working situations become more complex, which is that case for advanced robotic systems, using a framework with highly granular categories can be less effective. In our analysis, not all categories were represented. This does not necessarily indicate that these categories hold no importance to the automation of physical tasks through robotic systems, but more so that these are currently neither at the forefront of workers expectations nor the primary focus of research. In order to better represent the statements analysed in this study, researchers decided to combine the categories “*task design and function allocation*” as well as “*interaction design, operation and supervision.*” Future analysis may have a greater benefit from using the primary categories (*physical*, *psychosocial* and *organisational*) and then derive their own sub-categories, while using publications like the OSH-factors framework for advanced robotics as a guideline (Rosen et al., 2022).

### 6.2 Positive—neutral—negative expectations

When comparing the initial expectation of change towards the robotic system, as displayed in Table 3, one can observe two general tendencies. Firstly, the replies heavily lean towards positive changes. The most frequently named expectations related to unspecified changes was “*overall improvement of the work situation.*” This goes along with the second most named category, namely, “*improved physical ergonomics.*” This was as frequently addressed as the expectation for the robotic system to increase the efficiency in production. Other positive changes that were also named were a reduction in work intensity, an increased task variability and that the introduction will lead to an upskilling of workers. The initial positive expectations align with the impact robotic systems typically have on a workplace according to literature (improved efficiency (Evjemo et al., 2020), and ergonomic improvements (Colim et al., 2020). Looking at the neutral and negative responses, workers either expected a general increase of their work, explicitly express that they are uncertain what will change, or doubted the applicability of robotic systems at their workplace. This might indicate a lack of knowledge about automation, the capabilities or intended uses. Overall, we see a similarly mixed distribution of expectations as in previous studies on this topic (Wurhofer et al., 2015; Kildal et al., 2018) with a slight lean towards positive change.

The technology as well as public perception and media reporting on it, may have changed over time, influencing workers’ answers (Riemer and Wischniewski, 2019). While it is not possible to conclusively determine the reason why this sample’s initial expectations were more positive than in prior studies (Wurhofer et al., 2015), it underlines that it is valuable to assess these expectations in workers. Not only to gauge if their expectations are realistic, but also to identify any distrust or fears related to the technology, as these can have a negative influence on the implementation process and later use of the robot (Hancock et al., 2011; Marangunić and Granić, 2015).

### 6.3 Opportunities and risks on a time frame

The follow-up questions of the general change workers expected from the technology were targeted at short-term as well as long-term risks and benefits. Literature shows that people consider distant or immediate consequences of potential behaviours or events differently (Strathman et al., 1994). Moreover, the Construal-Level Theory of Psychological Distance states that the further removed something is from direct experience, the more abstract the level of construal of the matter (Trope and Liberman, 2010). Hence, results should indicate a greater level of detail in the expected short-term changes, compared to the long-term consequences, which matches our findings.

#### 6.3.1 Short- and long-term changes

When workers were asked to give specific examples on short-term opportunities and risks, they provided a variety of answers with varying depth. The most named opportunity was an overall improvement of the work situation without any further specifications, followed by the expectation that the robot will improve physical ergonomics. However, a number of other OSH related factors were named in greater detail. From the named opportunities (Table 2), the workers’ expectations for the system in the short-term were that it will benefit their working conditions by alleviating both physical and mental strain. Workers were able to formulate their short-term expectations in great detail. Workers expect the robot to have errors or produce work at a lower quality. There is minor concern about the physical safety of the system but a stronger focus on the machine taking up too much space in the current workplace. When OSH related factors were named by workers, they focus on psychosocial factors like increased stress, unclear task allocation or a low acceptance for the technology which literature shows can spike during the early days of use (Wisse and Sleebos, 2016; Tuomi et al., 2021). We also see that a lack of training is mentioned in the short-term, which could potentially contribute to the expected errors and in the long run, job loss. Interestingly the short-term risks indicate that while workers are aware that the robotic system will alter their physical workspace and has residual physical risks, they name negative psychosocial effect more frequently than physical.

Regarding the long-term changes, there were fewer risks than opportunities named and those exhibited a lower level of detail. Workers name primarily OSH related long-term opportunities, like an increased task variability, prevention of long-term health consequences and the improvement of both physical and cognitive ergonomics at the workplace, which aligns with literature findings (Kim et al., 2017; Kadir et al., 2019). The most dominant group here is the improvement of physical ergonomics. However, there were also contributions from individuals who expected opposing effects: an increase in task variability or more monotony. The most commonly named long term-risk was job loss; the fear of which triggered by automation at the workplace is well documented (Bhattacharyya, 2023). Malfunctions, too, were named as a long-term phenomenon of the technology, however to a lesser degree than in the short-term. Overall, the long-term consequences were formulated to a lower level of detail, which generally aligns with the Construal-Level Theory of Psychological Distance (Trope and Liberman, 2010).

This comparison of both short- and long-term risks and opportunities highlights that worker are well aware of the potential impact a robotic system can have on them and their work environment, not just imminently, but also over time. Long- and short-term expectations from workers towards robotic systems, OSH related and non-OSH related, is a highly under researched area. Few studies on the OSH impact take an explicit timeframe into consideration, with the exception of long-term physical strain effects like MSD (Haddadin et al., 2009). None of the above included publications specified the effects to a certain time frame.

## 7 Limitations

While great efforts were made, to uphold high scientific standards, some limitations still apply to the results of this research. The worker survey took place in their mother tongue, however the results had to be translated for further analysis. While a high standard of translation was aimed for, linguistic nuance was inevitably lost in translation. Furthermore, the surveyed workers had different levels of experience, specifically the German subsample, as they had already worked with the robot by the time the survey took place. This may have informed their replies to the survey. Although a large proportion of employees in the workplaces surveyed participated, the overall sample size is moderate. Regarding the analysis of short-term and long-term consequences, it has to be noted that some participants gave identical answers for both, leaving it open to interpretation if they expect the effect to be persistent, or to change over time.

## 8 Future research

The present study has provided a comprehensive examination of the multifaceted risks and opportunities associated with robotic systems in the context of workplace automation, particularly in industrial settings. However, to further enhance the depth and applicability of our findings, there is a need for future research. An important next step could be a validation of our findings through expert consensus assessment by involving experts in the fields of robotics, occupational safety and health (OSH), and industrial automation. By gauging the level of agreement among experts, it would be possible to ascertain whether our conclusions align with a broader expert consensus. Another research avenue that can be explored is, preforming the above demonstrated procedure in other sectors that are likely to see increased robot usage in the near future, like the agricultural or medical sector. This would allow a broader comparison between the sectors, possibly unveiling critical overlap or discrepancies between the expectation and OSH factors between the sectors.

Lastly, a topic which is continuously growing in relevance and prominence, when it comes to the integration of robotic systems into the world of work at large, as well as the industrial sector specifically, are the ethics and legislative challenges these technologies create. Their expanding capabilities in perceiving their work environment are already in focus of matters regarding data privacy and personal data collection. Future research should focus on the specific ethical challenges for the industrial sector as well as the world of work at large.

## 9 Conclusion

More and more workers are expected to interact with robotic systems at their workplace. In order to create a human-centred workspace, it is necessary to be aware of worker expectations as well as current research on the risks and opportunities these technologies may bring. In order to gain a better understanding of research results, both theoretical and from worker surveys, it can be helpful to use existing models or frameworks to create a common ground for analysis. The OSH-factors framework for advanced robotics divides the topic into physical, psychosocial and organisational facets. Central question to our paper was whether and how these OSH dimensions and effects apply for the automation of physical tasks through interactive robotic systems in the manufacturing sector, represented by literature as well as a worker survey. Furthermore, we analysed tendencies (positive or negative) in workers expectations in the long- and short-term, as this is a critically under researched topic. We found that the framework is applicable to the reviewed data with limitations. The three main categories could be applied to the statements with high interrater reliability, showing that they are suitable as a baseline for further analysis. Most of the subcategories provide additional nuance to that analysis. However, not all subcategories are distinct enough and show significant overlap. Combining the categories may help better represent the underlying data. There are several categories in the framework that are underrepresented, both in literature, as well as the worker survey. Especially the lack of focus on the introduction process offers potential for future research.

Regarding expected short- and long-term changes, both the positive and negative details are prevalent expectations. Both short- and long-term opportunities focus on physical ergonomics, however, they also contain detailed suggestions on how workers expect their jobs to change towards less monotonous work, and more control over their time and decision making. Short- and long-term risks were highly varied and addressed topic relating to physical, psychosocial and organisational aspects.

The results of the study highlight the predominantly positive impact of robotic systems on physical factors, including reduced physical strain, removal from unsafe work environments and long-term ergonomic improvement. From the literature perspective, there is a lack of long-term study results on the impact of these technologies. The interviews however indicate that workers do approach these technologies with the expectation of long-term health benefits. However, both the literature and the workers’ perspective also identified potential psychosocial risks, including an increase in cognitive demands and concerns about job loss.

Overall, this article provides insights for researchers, practitioners, and policymakers involved in the design and implementation of robotic systems in the workplace. While the results suggest an overall positive impact expectation of robotic systems on occupational safety and health in the manufacturing sector, it also highlights that workers expect negative changes to come from the technology. Further research is needed to assess long-term effects and ensure that workers’ wellbeing is prioritized in the process of automation.

## Data Availability

The raw data supporting the conclusion of this article will be made available by the authors, without undue reservation.
